# The Content of Elements in Infant Formulas and Drinks Against Mineral Requirements of Children

**DOI:** 10.1007/s12011-014-9947-1

**Published:** 2014-04-06

**Authors:** A. Molska, I. Gutowska, I. Baranowska-Bosiacka, I. Noceń, D. Chlubek

**Affiliations:** 1 Department of Biochemistry and Human Nutrition, Pomeranian Medical University, Broniewskiego Str. 24, 71-460 Szczecin, Poland; 2 Department of Biochemistry, Pomeranian Medical University, Powstańców Wlkp. av. 72, 70-111 Szczecin, Poland; 3 Department of Medical Chemistry, Pomeranian Medical University, Powstańców Wlkp. av. 72, 70-111 Szczecin, Poland

**Keywords:** Fluoride, Calcium, Magnesium, Zinc, Iron, Infant formulas

## Abstract

The present study aimed at analysing the content of fluorine (F), calcium (Ca), magnesium (Mg), iron (Fe) and zinc (Zn) in the drinks for children and infant formulas, a popular supplement or substitute for breast milk produced from cow milk on an industrial scale. Ca, Mg, Zn and Fe concentrations were determined using atomic absorption spectrophotometer, while F levels using a potentiometric method. F levels in the examined formula samples increased with the intended age range, until the intended age of 1 year, and then decreased. A lower content of Ca, Mg and Zn was observed in formulas intended for children <1 year of age and higher for older children. Fe content increased with the age range. A statistically significant higher content of Ca, Mg, Zn and Fe in samples intended for children with phenylketonuria in comparison to those intended for healthy children or children with food allergies was noted. The content of the analysed elements in juices and nectars showed the highest contents in products intended for infants (under 6 months of age). The lowest levels of elements tested were found in drinks for children over 6 months of age. In conclusion, the concentrations of the examined elements in infant formulas and juices for children were decidedly greater than the standards for the individual age groups. Although the absorption of these elements from artificial products is far lower than from breast milk, there is still the fear of consequences of excessive concentrations of these minerals.

## Introduction

Infant formulas are a popular supplement or substitute for breast milk. Produced from cow milk on an industrial scale, the formulas are divided into two basic types: (i) products for newborns and (ii) for children older than 4 or 6 months [1]. The world’s leading experts in the field of infant nutrition, working in the ESPGAN Nutrition Committee and the EU Scientific Committee on Food, established standards for formulas for healthy children, with a detailed content of 11 minerals and 13 vitamins (Directive 91/321/EEC of 14 May 1991 and amended 16 February 1996 (Dir. 96/4/EC)) [2].

In addition to formulas for healthy children, there are also special preparations for children with phenylketonuria (PKU), one of the most common newborn defects in amino acid metabolism. This inherited autosomal recessive disorder causes a deficiency of phenylalanine hydroxylase (PAH) [3]. The diet of infants is also often complemented by granular fruit and herbal teas, which may be given to infants after 4 months of age, and juices and nectars which are rich in valuable macro- and micro-nutrients. The latter may be used as first food products other than milk or formula that can be introduced to the baby’s diet [4]. They may enrich the child’s diet with additional quantities of elements.

Minerals are indispensable in human nutrition, and their content in the body depends on their occurrence in soil, drinking water, and nutrition [5]. A deficiency and excess of any chemical elements may induce adverse effects in the human body [6, 7], especially in children [8]. This is true even in the case of calcium, an elevated supply of which may lead to severe kidney damage [5], while acute shortages can cause excessive excitability of the nervous system, parathyroid hyperplasia, changes in the intestinal flora, and even decalcification and deformation of bones [9, 10].

For these reasons, it is important to determine the quality and contents of food products for children, including formulas, teas, juices and nectars, in terms of the daily doses of macro- and micro-nutrients [11].

## Material and Methods

The material for this study involved formulas (powder or tablets), teas (granulate) and juices and nectars intended for children of various ages. Analysis involved a total of 46 samples: 23 infant formulas, 3 teas, 9 nectars and 11 juices.

We specified the form of administration, the age of children for whom the product was designed, and diseases in which the product could be used without harm (PKU, food allergies, colic, constipation).

### F Determination

Tablets and granules were triturated in an agate mortar. Test portions of about 10 mg were quenched with 1 ml of boiled double-distilled water at a temperature of 50 °C and then vortexed. Samples of juices and nectars were collected directly from bottles, after thorough mixing of the contents.

To a 0.5-ml solution sample, we added 0.5 ml of TISAB II buffer, and after 5 min, the potential of the samples was measured by potentiometry using a Thermo Orion ion-selective electrode (Thermo Scientific, USA). Then, 0.1 ml of the appropriate standard was added and measurement was performed again. The electrode had been calibrated using standard solutions.

### Ca, Mg, Fe and Zn Determination

Powdered samples of formulas and teas (about 10 mg each) were collected into plastic tubes, and 5 mL of double-distilled H_2_O was added. Liquid samples of juices and nectars (1 ml) were also diluted in 5 ml of double-distilled H_2_O. Calcium, magnesium, iron and zinc were determined with an atomic absorption spectrometer which had been calibrated using standard solutions. Determinations were made in an air-acetylene flame against the corresponding lamps at the following wavelengths: Ca, 422.7 nm; Mg, 285.2 nm; Fe, 248.3 nm; and Zn, 213.9 nm.

### Statistical Analysis

Obtained results were analysed statistically using the software package Statistica 10, StatSoft, Poland. Arithmetical mean and the standard deviation (SD) were found for each of the studied parameters. The distribution of results for individual variables was obtained with the Shapiro-Wilk *W* test. As most of the distributions deviated from the normal Gaussian distribution, non-parametric tests were used for further analyses. Correlations between the changes of the parameters were examined with the Spearman’s rank correlation coefficient. To assess the differences between the studied groups, the non-parametric Mann-Whitney test was used. The level of significance was *p* ≤ 0.05.

## Results

**Fig. 1:**
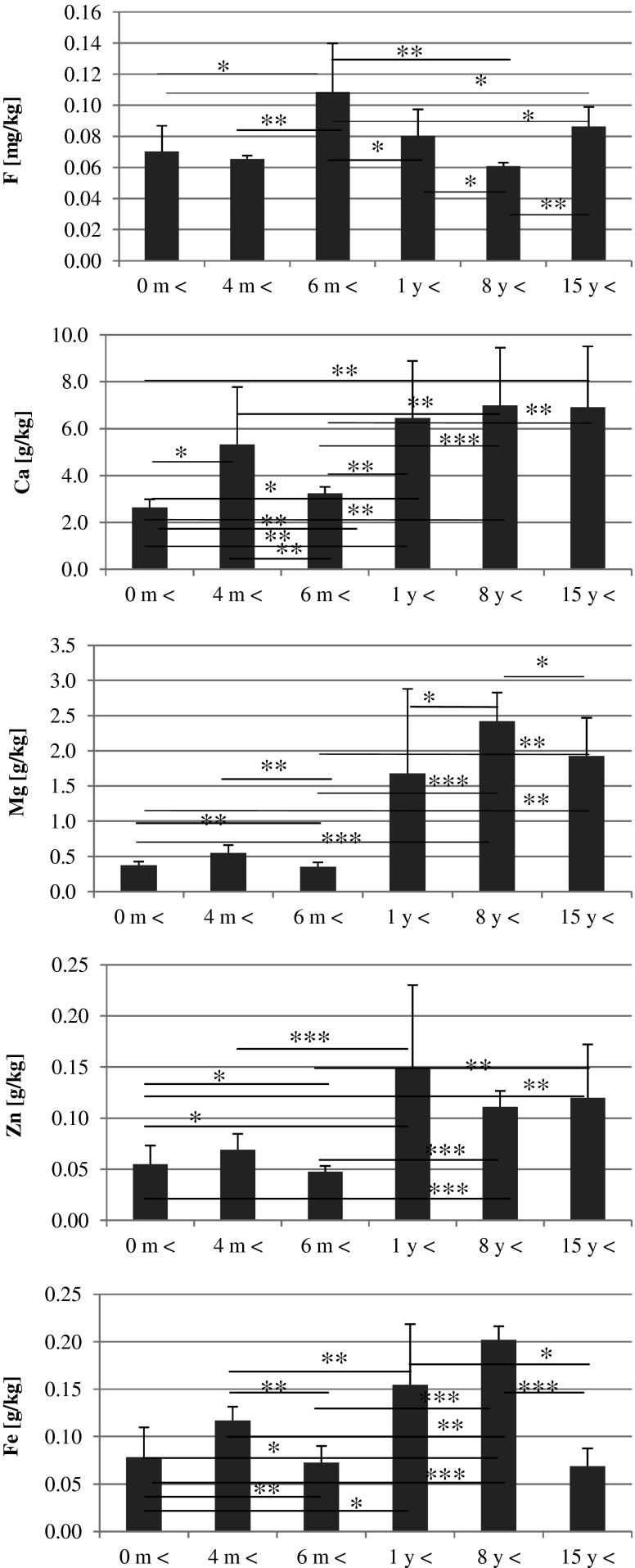
Age-dependent elements content in the infant formulas. Statistically significant differences at *p > 0.05, **p > 0.01, and ***p > 0.001

**Fig. 2:**
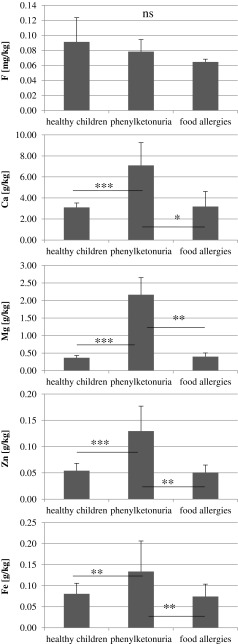
Destination-dependent elements content in the infant formulas. Statistically significant differences at *p > 0.05, **p > 0.01, and ***p > 0.001

**Fig. 3:**
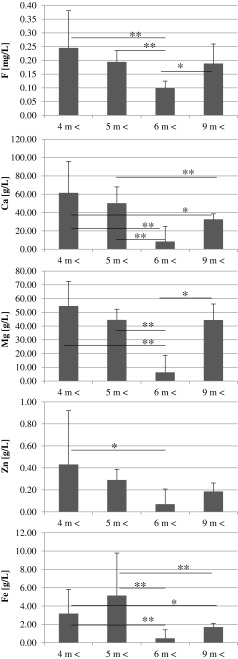
Age-dependent elements content in the drinks for children. Statistically significant differences at *p > 0.05, **p > 0.01, and ***p > 0.001

In the samples of food products intended for children, we determined the levels of five elements: fluorine, calcium, magnesium, zinc and iron. The results were then divided into groups according to (1) the intended age range, (2) the child health status appropriate for the consumption of the product, and (3) the form of administration. Then, the results were subjected to statistical analysis, and the results were placed into graphs (Figs. 1, 2 and 3).

### Infant Formulas

Fluorine levels in the examined formula samples increased with the intended age range, until the intended age of 1 year, and then decreased. The highest fluorine level was observed in formula samples for children of at least 6 months old.

With regard to calcium, magnesium and zinc, a lower content of these elements was observed in formulas intended for children <1 year of age. Formulas for older children contained significantly higher contents of these elements.

The highest Ca and Mg levels were observed in samples for children over 8 years of age, and the highest Zn levels were found in formulas for children over 1 year of age.

Fe content in the formula samples increased with the age range and reached the highest value in products for children above 8 years of age (Fig. 1).

Spearman rank correlation analysis showed significant positive correlations between Ca and Mg (*r* = 0.88, *p* < 0.0000), Ca and Zn (*r* = 0.81, *p* < 0.0000), Ca and Fe (*r* = 0.44 *p* < 0.0088), Mg and Zn (*r* = 0.84, *p* < 0.0000), Mg and Fe (*r* = 0.56, *p* < 0.0006), and Zn and Fe (*r* = 0.68, *p* < 0.0000).

Analysis of formulas in terms of diseases for which they were intended to be consumed demonstrated a statistically significant higher content of calcium, magnesium, zinc and iron in samples intended for children with phenylketonuria in comparison to those intended for healthy children or children with food allergies.

Fluorine content was highest in formulas for healthy children and lowest for children with food allergies, but the differences were not statistically significant (Fig. 2).

### Juices and Nectars

The content of the analysed elements in juices and nectars showed the highest contents in products intended for infants (under 6 months of age). The lowest levels of elements tested were found in drinks for children over 6 months of age (Figs. 1, 2 and 3).

Spearman rank correlation analysis showed significant positive correlations between the contents of F and Ca (*r* = 0.68, *p* < 0.0003), Ca and Mg (*r* = 0.43, *p* < 0.04), Ca and Fe (*r* = 0.44, *p* < 0.03), Mg and Zn (*r* = 0.53, *p* < 0.009), Mg and Fe (*r* = 0.60, *p* < 0.002), and Zn and Fe (*r* = 0.75, *p* < 0.0000) in the juices and nectars intended for children.

## Discussion

Infant formulas must meet very high quality standards, ensuring complete safety of their use. They contain well-defined proportions of nutrients such as lipids, proteins, carbohydrates, vitamins and minerals. The aim is to ensure that their composition maximally resembles mother’s milk, and to this purpose, producers try to improve the proportions of protein and fat, increase vitamin and mineral content, and also add probiotics, prebiotics and omega-3 fatty acids [12, 13].

## Calcium

Calcium is contained in bone tissues and some types of cell walls. Its total content in the human body is 1.38 %, of which 99 % is bone hydroxyapatite [14]. Calcium requirements differ for different groups of people and depend on gender, age and physical activity. In addition to the above, calcium supply is very important in pregnancy, particularly in the third trimester when the fetus intakes from 25 to 30 g of calcium [15]. Also, after pregnancy, a mother must provide a child with adequate amounts of this element together with her milk, where its concentration is about 0.33 g/kg (33 mg/100 ml), considerably less than in infant formulas [2], where the high concentration of this element comes from the cow milk [11] used to produce formulas. Another reason is the lower bioavailability of this element from formulas (38 %) compared with mother’s milk (58 %) [16]. According to Panczenko-Kresowska et al. [5], the recommended daily norm of calcium consumption for children over 1 year of age is 800–1,000 mg/person, which is based on the composition of mother’s milk [17]. Compared to the recommended dose, the tested products showed an almost six times higher level of calcium. Although the bioavailability of calcium from formulas is lower, there is a risk of exceeding the daily intake of this element, which in turn can cause damage to kidneys and impair the absorption of magnesium, iron and zinc [5].

### Magnesium

Magnesium is essential for the proper functioning of the body. It decides the correct course of pregnancy [18], and in newborns, Mg deficiency may lead to the sudden infant death syndrome [18, 19]. Breast milk, with a Mg content of 34 mg/l, is the best source of this element for infants [5, 17]. Research by Ziegler et al. [20] showed that a newborn in the initial stage of life intakes, on average, 30 mg Mg/day from the mother’s milk, and at 6 months of age, this quantity reaches 40 mg/day. Magnesium metabolism is closely associated with calcium metabolism. In the human milk, the ratio of calcium to magnesium is 9:1 [2], while in the examined formulas, it was 4:1. This increased magnesium content may result from a smaller absorption of this element from formulas compared to mother’s milk [5]. In this situation, it appears that Mg deficiency in children fed with formulas is rather unlikely. However, exceeding the recommended Mg doses as a result of supplementation with large amounts of child juices and teas could result in nausea, vomiting and hypotension [21].

### Fluorine

Experts believe that the intake of fluorine for infants from birth to 6 months of age should be 0.1–0.5 mg/kg/day [5]. In infants fed with powdered formulas, the intake of this element is heavily influenced by the level of F in the water used in preparation [22]. If the level exceeds 0.7 mg F/l, the possibility of adverse changes in the body increases [23, 24], including symptoms such as fluorosis of enamel and bone [25]. The maturation of enamel is a period with the greatest sensitivity to the effects of fluorine. For the front fixed teeth, this period covers the 22nd–25th months of human life [26]. F deficiency may contribute to aberrant mineralization of hard tissue, leading to the development of caries and osteoporosis [27].

In March 2013, scientists from South Korea, in cooperation with the University of Boston, started research on food for infants up to 6 months of age [28]. In an analysis of 28 different samples of infant formulas, it was estimated that when the formulas were mixed with water containing 0, 0.5, 0.8, and 1.0 mg F/kg, infants consumed a total of 0.018 to 0.298 mg F/kg/day [28], which greatly exceeded the recommended standards [22]. Importantly, additional supplementation with ready-made juices and teas may further increase the intake of fluorine.

### Iron

Significant iron deficiency in infants may lead to impaired cell-mediated immunity, while an excess may lead to serious liver damage [29, 30]. With optimal reserves of Fe in the newborn, the absorption of this micronutrient supplied with breast milk after birth is sufficient to cover the daily requirements of children [31, 32]. At about 4–5 months of age, an increasing demand of tissues for Fe increases to about 0.5 mg/day [5]. Any formulas should therefore be supplemented with Fe, and after premature delivery, additional Fe administration should start from 2 to 3 months of age [2]. Unfortunately, Fe deficiency in infants is common, with the most important risk factors including low birth weight, early introduction of cow’s milk, and rapid growth [33]. In research by Marzec et al. [34], Fe level was measured in the basic foodstuffs for infants and children per day. Assuming that a year-old child weighing about 10 kg consumes one carton of juice daily (about 200 g), one package of soup (about 170 g) and 1 package of dinner (about 185 g), then the child eats 1.68 mg Fe/day, which represents only 11 % of the daily requirement and may result in Fe deficiency [34, 35]. However, our study showed high concentrations of this element in the formulas and fruit juices that may complement deficiency of this element in the body.

### Zinc

Zinc, described by many authors as a 21st century element [36], is required for the synthesis of protein and nucleic acid metabolism [2]. Long-term studies on monkeys fed a diet without zinc showed disturbances in the metabolism of vitamin A [5, 37] and in the formation of the skeletal and immune systems in the fetus [37]. For infants up to 1 year of age, the recommended daily amount of Zn is 5.0 mg/day, and in further development, it increases, depending on the sex of the child [36]. The concentration of this element in natural mother’s milk is about 2 mg/kg and decreases over time with lactation [2]. After 3 weeks, it is only 36–54 % of the initial concentration, and after 3 months, not more than 20 % [2]. In the present study, in the examined infant formula samples, the amount of this element was more than ten times higher than in human milk, which may be associated with the poor absorption of zinc from artificial products. Despite this, there are concerns of exceeding the acceptable daily intake while feeding the child with fruit juices and teas. An excess of zinc can lead to microcytic anemia or neutropenia and reduce the concentration of iron in the body [5]. Additionally, zinc competes with magnesium at the absorption level in the intestines and in the structural parts of the bone [38].

## Conclusion

In conclusion, the concentrations of the examined elements in infant formulas and juices for children were decidedly greater than the standards for the individual age groups. Although the absorption of these elements from artificial products is far lower than that from breast milk, there is still the fear of consequences of excessive concentrations of these minerals.
